# Artificial intelligence and its clinical application in Anesthesiology: a systematic review

**DOI:** 10.1007/s10877-023-01088-0

**Published:** 2023-10-21

**Authors:** Sara Lopes, Gonçalo Rocha, Luís Guimarães-Pereira

**Affiliations:** 1 Department of Anesthesiology, Centro Hospitalar Universitário São João, Porto, Portugal; 2 Surgery and Physiology Department, Faculty of Medicine, University of Porto, Porto, Portugal

**Keywords:** Artificial Intelligence, Anesthesiology, Deep learning, Neural networks

## Abstract

**Purpose:**

Application of artificial intelligence (AI) in medicine is quickly expanding. Despite the amount of evidence and promising results, a thorough overview of the current state of AI in clinical practice of anesthesiology is needed. Therefore, our study aims to systematically review the application of AI in this context.

**Methods:**

A systematic review was conducted according to the Preferred Reporting Items for Systematic Reviews and Meta-Analyses (PRISMA) guidelines. We searched Medline and Web of Science for articles published up to November 2022 using terms related with AI and clinical practice of anesthesiology. Articles that involved animals, editorials, reviews and sample size lower than 10 patients were excluded. Characteristics and accuracy measures from each study were extracted.

**Results:**

A total of 46 articles were included in this review. We have grouped them into 4 categories with regard to their clinical applicability: (1) Depth of Anesthesia Monitoring; (2) Image-guided techniques related to Anesthesia; (3) Prediction of events/risks related to Anesthesia; (4) Drug administration control. Each group was analyzed, and the main findings were summarized. Across all fields, the majority of AI methods tested showed superior performance results compared to traditional methods.

**Conclusion:**

AI systems are being integrated into anesthesiology clinical practice, enhancing medical professionals’ skills of decision-making, diagnostic accuracy, and therapeutic response.

**Supplementary Information:**

The online version contains supplementary material available at 10.1007/s10877-023-01088-0.

## Introduction

Artificial Intelligence (AI) refers to computer science that enables machines to think and act rationally and it has become a part of the scientific development of many areas, including Medicine and particularly Anesthesiology [1].

AI employs a variety of theories, algorithms, and computational resources to carry out intelligent tasks with minimal human intervention, including decision-making, data analysis, complex problem-solving, event prediction, speech recognition, and visual perception [2]. It is believed that when data extraction, storage, access, and quality processes have been fully optimized, the potential of AI techniques will be able to drive a new technological paradigm, making it an excellent tool to apply on medical areas since it involves large volumes of biometric data with highly complex interrelationships [3].

AI includes several sub areas that are capable of extracting knowledge from a large dataset faster and more accurately than traditional methods, including machine learning, deep learning and robotics. Machine learning can analyze an extensive quantity of information and create an algorithm or model to detect patterns and perform prediction tasks without explicit instructions [4]. Neural networks, also known as artificial neural networks (ANNs) are a subset of machine learning and the basis of deep learning algorithms. It distinguishes itself because of the multiple layers that allow this technology to simulate the behavior of the human brain, making it possible to learn from multiple data and optimize accuracy. As for robotics, they stand for a mechanical system that is capable of interacting with the environment, automating tasks and offering pertinent recommendations based on the clinical scenario to aid decision-making [5] There are multiple applications of robotics in anesthesia, especially in conscious sedation using closed loop systems, but as well for the maintenance of anesthesia, hemodynamic management or to support decision making [6].

Searches in medical databases show that the amount of literature on AI is expanding quickly, indicating a remarkable academic focus in this field. There are several articles where different AI methods are successfully applied in screening, diagnostic and therapeutic techniques in various specialties [7–10]. Anesthesiology could benefit from this application as it is an area which requires clinical decision based on several continuous real-time variables. In this field, existing literature can be grouped into subareas concerning their clinical application, namely: depth of anesthesia monitoring, visually guided techniques using computer vision, prediction of risk of events during and after anesthesia, and control of anesthesia.

According to researchers, the next generation of doctors will need to be familiar with machine-learning methods for large data analysis [11]. It is crucial for clinicians in all specialties to understand these technologies and realize how to use them to provide safer, more effective, and more affordable treatment as the development and deployment of AI technology in medicine continue to expand [12].

Despite the exponential amount of evidence and promising results, a thorough overview of the current state of AI in anesthesiology clinical practice is lacking. There is a clear need to summarize the existing evidence in the form of a systematic review capable of serving as a guide.

This study aims to systematically review the application of AI methods in anesthesiology clinical practice of anesthesiology and discuss its future challenges and limitations.

## Methods

This systematic review was conducted in accordance with the Preferred Reporting Items for Systematic Reviews and Meta-Analyses (PRISMA) guidelines and registered on an international database of prospectively registered systematic reviews (PROSPERO) (CRD42023402952).

We searched Medline, through PubMed, and Web of Science for all English-language articles that were published up to November 2022 while using combinations of the following terms: “anesthesia, anesthesiology, artificial intelligence, neural network computer, machine learning, humans” (Table 1).

**Table 1:** Strategy search used in Medline and replicated in Web of Science

Search number	Query
#1	artificial intelligence [MeSH Terms]
#2	anesthesia [MeSH Terms]
#3	anesthesiology[MeSH Terms]
#4	machine learning[MeSH Terms]
#5	neural network computer[MeSH Terms]
#6 #7 #8	((neural network computer[MeSH Terms]) OR (machine learning[MeSH Terms])) OR (artificial intelligence[MeSH Terms]) deep learning [MeSH Terms] neural network [MeSH Terms]
#9	humans[MeSH Terms]
#10	(anesthesiology[MeSH Terms]) OR (anesthesia[MeSH Terms])
#11	review[Publication Type]
#12	editorial[Publication Type]
#13	comment[Publication Type]
#14	((comment[Publication table Type]) OR (editorial[Publication Type])) OR (review[Publication Type])
#15	#6 AND #8
#16	#13 NOT #12

Studies were included if the primary aim was the application of AI–based algorithms in clinical practice of anesthesiology. Articles that involved animals, editorials, reviews, sample size lower than 10 patients or had the inappropriate study design (including studies with inappropriate comparator, outcome, or setting, etc.) were excluded.

Two reviewers, GR and SL, screened articles for inclusion or exclusion using the online platform Covidence®. Each article was screened independently by two reviewers. Any disagreement among the two screeners would be solved by a third reviewer - LG. One reviewer extracted data from articles using a Microsoft Excel® spreadsheet, and the other one checked the extracted data. The extracted data included study aim, study design, AI method used, control or/and comparator used, number of population studied, measures of effect and main conclusions.

The Joanna Briggs Institute (JBI) critical appraisal checklist for analytic cross-section and case-control studies were used to assess the risk of bias of studies. The risk of bias was rated according to the percentage of positive items in the checklist: low (higher than 70%) moderate (50–69%) and high (lower than 50%).

Due to variable design and methods of reporting results, a meta-analysis was not able to perform; as a result, the findings of the included papers were descriptively outlined.

## Results

**Fig. 1:**
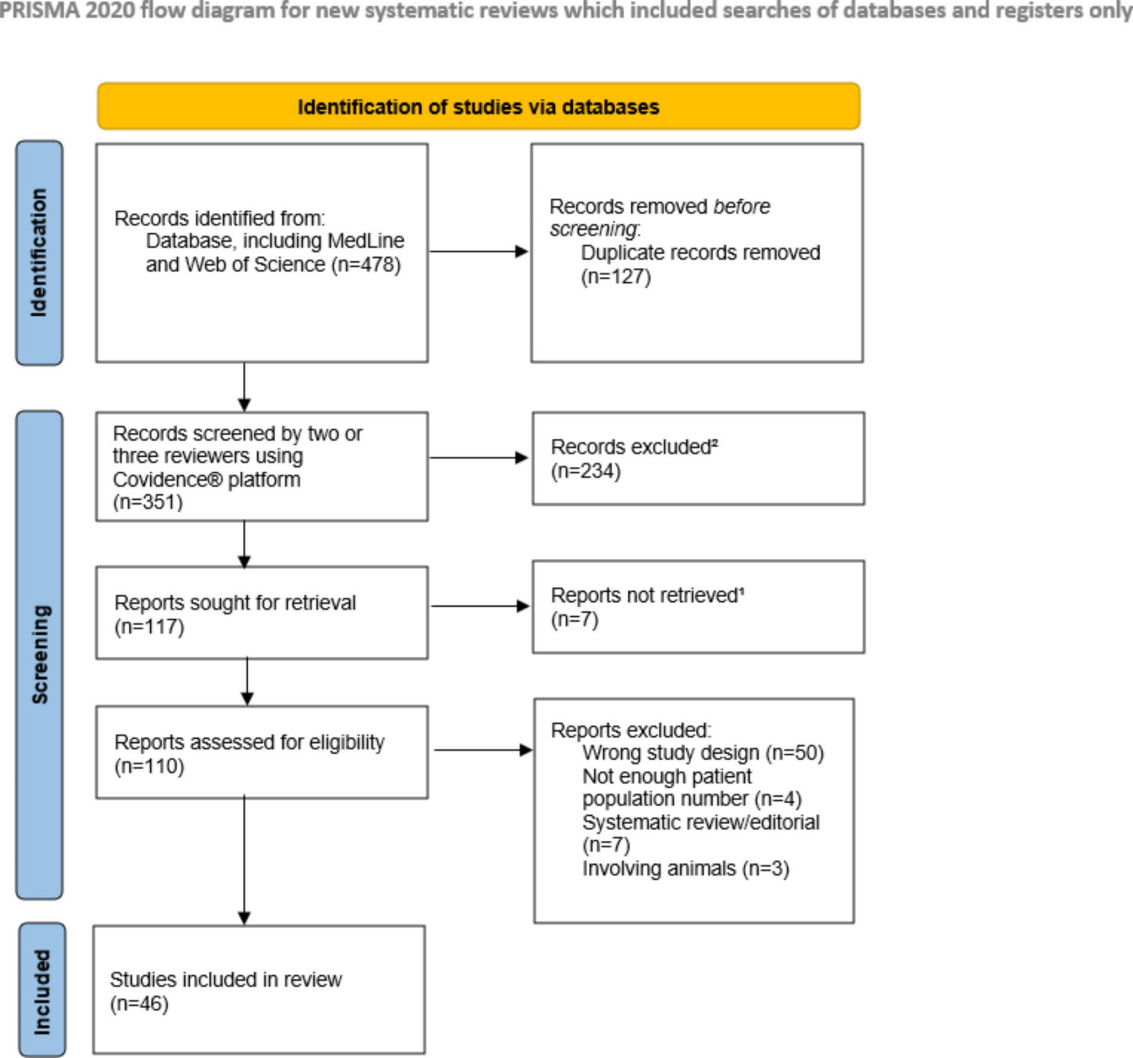
PRISMA flow chart outlining the selection of studies for review Ref: http://prisma-statement.org/prismastatement/flowdiagram.aspx ¹Articles which were unable to find the full text ²Not anesthesia related or without clinical applicability

A total of 478 records were identified from databases, 127 duplicates were removed. The remaining 351 articles were submitted to abstract screening and 234 were excluded. The last 110 reports were full-text reviewed and after the application of the exclusion criteria, 46 studies were selected for final analysis. Figure 1 shows the PRISMA 2020 flow diagram used for the selection of the articles.

We identified the risk of bias as low in 25 studies, moderate in 25, and high in 6 (Supplementary Information 1). The majority of papers with a high or moderate risk of bias lacked identification of confounding factors and solutions for addressing them.

In order to clarify the description and extraction of results from the articles, we have grouped them into 4 categories with regard to their clinical applicability: (1) Depth of Anesthesia Monitoring; (2) Image-guided techniques related to Anesthesia; (3) Prediction of events/risks related to Anesthesia; (4) Drug administration control.

Despite the wide variety of subjects covered, all of the articles founded shared a common goal: that of maximizing the newly discovered potential of AI approaches in order to enhance a variety of anesthesiologists clinical abilities and responsibilities.

### Depth of Anesthesia Monitoring (DoA)

We obtained 13 articles regarding the application of AI in DoA monitoring, as described in Table 2.

**Table 2:** The application of AI in DoA monitoring

Study	Aim	Population	AI method	n	Accuracy methods	Conclusions
Afshar 2021 [13]	Predict BIS values from EEG	Subjects who received vascular surgery	CNN	35	Accuracy 88,71%; Sn 77.62%; AUC 81,11%; RMSE 5.59; MAE 4.3 ± 0.87	The model outperforms the competitive methods particularly, for large EEG datasets.
Gu 2019 [33]	Using EEG signals to estimate different anesthetic states	Adult patients under general anesthesia with propofol	ANN	16	Sn 79.1%; CC between BIS, 0.892	This method is promising and feasible for a monitoring system to assess the DoA.
Jiang 2015 [14]	Using EEG signals and Sample Entropy analysis to estimate DoA	Aged 22–79 years under surgery with general anesthesia	ANN	24	CC, 0.73; MSE, 10.19; AUC, 0.953	Purpose method is closer to experienced anesthesiologists than BIS index.
Lee 2018 [34]	Predict BIS index	Patients who underwent general surgery under propofol and remifentanil	ANN	100	CC 0.561	The model predicted BIS more accurately compared to the traditional model
Madanu 2021 [35]	Predict DoA form EEG signals	Adults who underwent ear, nose and throat surgery	CNN	50	Accuracy of the best trained model 83.2%	The proposed method provides a robust and reliable benchmark for DOA level classification
Ortolani 2002 [36]	Index of DoA from processed EEG data	Adults, ASA I score, who underwent general abdominal surgery	ANN	50	CC with BIS, 0.94	The developed model correlates very well with the BIS during anaesthesia with propofol.
Ranta 2002 [37]	Predict awareness with recall from intraoperative data	Adult patients under general anesthesia	ANN	544	Sn 23%; Sp 98%, Pk 0.66	A prediction indicating awareness by the network is very suggestive of true awareness and recall.
Shalbaf 2018 [38]	Predict state of DoA (qualitatively) from EEG features	Patients who underwent propofol and volatile anesthesia	Neuro-Fuzzy System	67	Accuracy, 92% in sevoflurane database; 93% propofol database	This method is applicable to a new real time monitoring system to assessment of DoA accurately.
Shalbaf 2020 [39]	Distinguish four levels of anesthesia from EEG data	Adults scheduled for orthopedic, gynecological or general surgery	SVM	17	Accuracy, 94.11%	The model is able to estimate level of hypnosis into awake, light, general and deep anesthetic states.
Tacke 2020 [15]	Index that predicts responsiveness from EEG and AEP signals	ASA status of I or II who underwent elective operations	SVM, ANN, Bayesian Algorithms	39	Highest PK (prediction probability), 0.935	The models can successfully develop an improved combined EEG and AEP parameter to predict DoA.
Zhan 2021 [16]	Heart rate variability to distinguish different anaesthesia states	Adult patients under general anaesthesia	DNN	23	Accuracies: 90.1% (DNN), 86.2% (LR), 87.5% (SVM) and 87.2% (decision tree)	The model could accurately distinguish between different anaesthesia states.
Zhang 2001 [40]	Predict states of anesthesia from AEP	Aged 21–77 years scheduled for elective abdominal surgery	ANN	21	Best identification accuracy was obtained using the five latencies model	AEP has useful information for identifying different anesthetic states.
Tosun 2010 [41]	Determining DoA during maintenance period for estimate anesthetic gas level	Patients undergoing general anesthesia with sevoflurane maintenance	ANN	250	Average accuracy 94%	The design system had successful results in the prediction of the anesthetic gas according to the anesthesia level.

*EEG* electroencephalography, *AEP* Auditory evoked potential, *BIS* bispectral index monitoring, *ANN* artificial neural networks *CNN* convolutional neural network, *SVM* support vector machine, *AUC* area under curve, *Sn* sensitivity, *Sp* specificity, *CC* correlation coefficient, *PK* Prediction Value, *MSE* Mean square error, *RMSE* Root mean square error, *MAPE* Median absolute error

Most of them focus on efforts to find a new DoA monitoring index capable of improving the acuity of the current means.

Our research has shown that most of the literature on this topic uses electroencephalograms (EEG) signals as input to an ANN as the preferred AI method for the purpose of estimating DoA. Due to its wide use on anesthesia, bispectral index (BIS) was mainly used as a control or comparator of the studies to assess the effectiveness of the chosen model, as shown in detail in Table 2.

*Afshar et al.* [13] proposed a new deep learning structure that uses multiple features from 35 patients EEG signals to continuously predict the BIS value, achieving an accuracy of 88.71% and an improvement in area under the curve (AUC) of 15% on average, when compared to traditional DoA estimation methods. On a different approach, *Jiang et al.* [14] uses EEG signals, pre-analysed through sample entropy as an input to train an ANN model that tries to provide a valuable reference to DoA. What sets the article apart from the rest of the subgroup is having used a score based on the clinical opinion of five experienced anesthesiologists – Expert Assessment of Conscious Level (EACL) - as the gold standard, contrary to most literature that tends to use the BIS index as the control. This allows the obtained model performance to be compared to the BIS itself. The results show that the mean correlation coefficient of the proposed model versus EACL on testing data is 0.73 ± 0.17, while the results of BIS index versus EACL are only 0.62 ± 0.19. This means that the proposed model is not only successful in estimating the anesthetic state, but also, does it more similarly to the clinical consensus than the BIS itself.

Whether the combination of different sources of clinical monitoring, in addition to the information normally collected from the EEG, could benefit the discriminative capacity of a DoA predictor algorithm is questioned by *Tacke et al.* [15]. To do so, they collect EEG and auditory evoked potentials (AEP) parameters used to test and compare the predictive power of several different AI methods, including support vector machine (SVM) and ANN, and given a different number and set of inputs. In this article, an algorithm was specially created with the objective of evaluating each parameter collected from the EEG signal (Spectral Entropy, Permutation entropy etc.) and AEP (Wavelet coefficients, amplitudes and latencies of wavelet, signal energies based on wavelet coefficients etc.) and define each predictive utility value, in a way the best set of parameters is defined. The findings demonstrate that among all the algorithms considered, SVM produces the highest prediction probability (PK) and, as supposed, the EEG and AEP parameter combination performs better than both “pure” parameter sets. The highest PK values produced with algorithms utilizing only AEP or EEG-parameters were 0.880 +/- 0.14 and 0.916 +/- 0.11, respectively, while the highest value with the combination of both was 0.935 +/- 0.11.

This is corroborated by *Zhan et al.* [16] who used four parameters (including the HRV high-frequency power, low-frequency power, high-to-low-frequency power ratio, and sample entropy) extracted from 23 patient electrocardiograms to predict their anesthetic state. The accuracy of the model was 90.01%, using the clinical evaluation of five anaesthesiologists as control.

### Image-guided techniques related to Anesthesia

Our search found 8 articles where AI techniques are applied to improve image-guided techniques in anesthesiology, as described in Table 3.

**Table 3:** The application of AI in image-guided techniques

Study	Aim	AI method	N	Acuraccy results	Conclusions
Alkhatib 2019 [42]	Median and sciatic nerve tracking in ultrasound images.	CNN	42	Accuracy, 0.87	Models showed superior performance and handled noise suppression without pre-filtering the images
Hetherington 2017 [17]	Identify lumbar vertebral levels in US images and display it with augmented reality.	CNN	20	Accuracy, 85%	The system successfully identifies lumbar vertebral levels.
InChan 2021 [18]	Determine the needle insertion point in obese patients using US image.	Machine-learning	48	Success rate for spinal anesthesia on first attempt 79.1%	The program is able to provide assistance to needle insertion point identification in obese patients.
Liu 2021 [20]	Locate, from US images, the anesthesia point of patients with regional nerve block.	CNN	100	Higher positioning accuracy and lower postoperative complications	The model can effectively improve the accuracy of US images.
Pesteie 2018 [43]	Automatically localize the needle target for epidural needle placement in US of the spine.	CNN	20	Average lateral and vertical error 1 mm, 0.4 mm, respectively.	The algorithm average errors are inferior to the clinically acceptable error.
Yoo 2021 [21]	Interpret video bronchoscopy images of the carina and main bronchi	CNN	180	Accuracy, 0.84; AUC 0.9752	This model can be a basis for designing a clinical decision support system with video bronchoscopy
Yu 2015 [44]	Identify the bone/interspinous region for US images obtained from pregnant patients.	SVM	20	Accuracy, 93.2 PPV, 94.17 Sensitivity, 93.05 AUC 97.55	Proposed method can process the ultrasound images of lumbar spine in an automatic manner.
Yusong 2016 [19]	Determine the needle entry site for epidural anesthesia in real time	SVM	53	Accuracy 0.94	Even the anesthetists with little experience in US could determine the suitable puncture site accurately and efficiently.

*US* ultrasonography, *CNN* convolutional neural network, *SVM* support vector machine

Convolutional neural networks (CNNs) were used to help identify important features in ultrasonography (US) imaging which is an extremely helpful tool in anesthesia, used for peripheral nerve blocks, point of care assessment or vascular access. The majority of publications in this group aim to increase the precision of needle target identification for epidural anesthesia since the current clinical method of blindly manual palpation of the spine has an associated low accuracy. *Hetherington* et all [17] develop a CNN-based system to identify lumbar vertebral levels in ultrasound (US) images with an accuracy of 85%, while *InChan et all* [18] specified this identification in a particular challenging group, obese patients (with an index of body mass superior than 30 kg/m2), with a success rate on first attempt of 79.1%. On a similar approach, *Yusong et al.* [19]*l* obtained the higher accuracy (0.94) of the group with a support vector machine model that determine the needle entry site for epidural anesthesia (EA) in 53 volunteer patients. The system guides the anesthesiologists to rotate and modify the position of the ultrasound probe to find the ideal puncture site. According to the findings, even anesthesiologists with limited experience in interpreting ultrasound images could find the ideal puncture site quickly and accurately.

Since it is a practical, safe, efficient, and affordable option, ultrasonography is used in many anesthesiology techniques, however, US images are frequently challenging to interpret because they are frequently affected by artifacts and shadowing. *Liu et all* [20] design a deep learning model to guide, from US images, the anesthesia of 100 patients with scapula fracture who underwent regional nerve block. The model is consisted of a CNN capable of image enhancement. The principle is classifying the image into two parts, the high-frequency and low-frequency components are separated, and different operations and processing are performed. In the end, the image is present with more detail and quality. It was used the traditional body surface anatomy for anesthesia positioning as the control group and the results showed that patients submitted to the system had higher positioning accuracy, better anesthesia effect, and fewer postoperative complications, with a significant difference.

CNNs are not exclusively applied to US images. *Yoo et all* [21] propose a CNN model to discriminate video bronchoscopy images of the carina and main bronchi regardless of rotation or covering. This system could be useful since orientation in the bronchial tree can often be confused and lead to accidental extubating or endobronchial intubation. The results were compared with 3 anesthesiologists and 3 pulmonologists with different time experiences and showed that the CNN model performed better (accuracy of 0.84) than nearly all human experts (0.38, 0.44, 0.51, 0.68, and 0.63) with only the most experienced pulmonologist displaying a similar performance (0.82).

### Prediction of events related to Anesthesia

A total of seventeen studies were found to use AI to predict anesthesia-related events. Six of them address post induction hypotension, three studies address hypoxia prevention, while the remaining ones span a wide range of other circumstances described on Table 4.

**Table 4:** The application of AI in prediction of events

Study	Aim	AI method	n	Accuracy results	Conclusions
**Prediction of Hypotension**					
Gratz 2020 [45]	Predict the likelihood of post spinal hypotension from arterial stiffness	ANN	45	AUC, 0.89; Sn, 0.84; Sp, 0.91	This study demonstrated that arterial stiffness variability is an effective predictor of postinduction hypotension.
Lin 2008 [46]	Identify patients with high risk of hypotension during spinal anesthesia	ANN	375	AUC, 0.796; Sn, 75.9%; Sp, 76.0%	The model should be useful in increasing vigilance in those patients most at risk for hypotension during spinal anesthesia.
Kang 2020 [7]	Predict postinduction hypotension from intraoperative data	Random Forest; ANN	222	AUC of Random Forest model 0.842; Accuracy 76.28%;	Models can predict hypotension occurring during the period between tracheal intubation and incision.
Kendale 2018 [47]	Prediction for the risk of postinduction hypotension	Gradient boosting machine	13.323	AUC 0.74	The model can forecast postinduction hypotension, with performance dependent on model choice and proper tuning.
Lin 2011 [48]	Identify patients at high risk for postinduction hypotension	ANN	294	Accuracy 82.3%; AUC 0.893; Sn 76.4%; Sp 85.6%;	The model has good discrimination of risk of postinduction hypotension.
Wijnberge 2020 [24]	Early warning system of hypotension during noncardiac surgery.	Machine Learning	68	Median time of hypotension 8.0 min intervention group vs. 32.7 in control group	The use of AI early warning system compared with standard care resulted in less intraoperative hypotension.
**Prediction of Hypoxemia**					
Geng 2019 [49]	Prediction of hypoxemia during sedation for gastrointestinal endoscopy	ANN	220	Accuracy 90%; AUC 0.80; Sn 14%; Sp 98%	The model was useful for prediction of hypoxemia.
Lundberg 2018 [26]	Predic the risk of hypoxemia and provides explanations of the risk factors.	Machine Learning	53.126	For initial prediction, AUC 0.83; For real-time prediciton AUC 0.81	The system can help improve the clinical understanding of hypoxemia risk during anaesthesia care.
Sippl 2017 [50]	Model perioperative hypoxia	ANN	124	Sn 74%; Sp 93%	The model is able to classify oxygen desaturation on a level similiar to the mutual agreement between human experts.
**Prediction of different events**					
Huang 2022 [51]	Prediction of surgery and anesthesia emergence duration	ANN	4.285	Accuracy, 0.9552	Prediction accuracies of the proposed serial prediction systems are acceptable in comparison to separate systems.
Huang 2003 [52]	Predict response during isoflurane anaesthesia from time series of EEGs	ANN	98	Accuracy 91.84%	The technique outperforms competing techniques, is computationally fast, and offers acceptable real-time clinical performance.
Knorr 2006 [53]	Distinguish between normal breathing and obstructed airway events.	ANN	10	Accuracy 86.1%; Sn 72.9%; Sp 93.0%	The model has potential to distinguishing between normal and obstructed airway events.
Mansoor Baig 2013 [54]	Detection of absolute hypovolaemia	Fuzzy Logic	20	Kappa value of the best FL model, 0.75	FLMS-2 model has shown to accurately detect differences between the levels of hypovolaemia
Peng 2007 [27]	Predict postoperative nausea and vomiting in patients who received general anaesthesia.	ANN	430	Accuracy, 83.3%; AUC, 0.814; Sn, 77.9%; Sp 85.0%	The ANN model appears to be a suitable model for clinicians to use cost-effective antiemetic treatments.
Ren 2022 [55]	Predict the amount of blood transfusion during cesarean section.	XGB classifier	150	Accuracy 0.953: AUC 0.881	The XGB model has a strong prediction performance, can offer precise individual predictions for patients, and has a promising future in clinical use.
Santanen 2003 [56]	Predict the recovery of a neuromuscular block during general anaesthesia	ANN	66	CC 0.91; Mean absolute prediction error 6.75	Model could predict individual recovery times significantly better than the average-based method.
Zhang 2018 [57]	Predicts a patient’s ASA using the patient’s home medications and comorbidities.	RF	41.932	AUC 0.884; Cohen’s Kappa 0.456;	RF algorithm can predict ASA with agreement identical to that of anesthesiologists described in literature.

*EEG* electroencephalography, *ANN* artificial neural networks *CNN* convolutional neural network, *SVM* support vector machine, *RF* Random Forest, *AUC* area under curve, *Sn* sensitivity, *Sp* specificity, *CC* correlation coefficient

*Kang et al.* [22] tested the effectiveness of machine learning models in predicting late post induction hypotension (PIH), defined as hypotension occurring from tracheal intubation to incision. The inputs to develop the model were, not only the clinical records of 126 patients, but also intraoperative monitoring data from the early anesthetic induction phase, such as general anesthesia monitor signals. The random-forest model performed best among the four studied systems (naive bayes, logistic regression, random forest, and ANN) with an area under the receiver operating characteristic curve of 0.842. Lowest systolic blood pressure, lowest mean blood pressure, and mean systolic blood pressure before tracheal intubation were the three factors that had the biggest impact on the accuracy of machine learning prediction. On a similar attempt, a neural networks model was used to identify patients with high risk of hypotension during spinal anesthesia (sensitivity of 75.9%; specificity of 76.0%; AUC of 0.796) and was found to exceed predictions of all five senior anesthesiologists (sensitivity 16.1 − 36.1%; specificity 64.0 − 87.0%) [23].

On a more practical and clinical-oriented approach, *Wijnberg et all* [24] made a randomized controlled trial to evaluate if a machine learning early warning system would reduce hypotension during noncardiac surgery. The algorithm uses 23 parameters extracted from an arterial pressure waveform measured continuously to detect deteriorations in cardiovascular compensatory mechanisms that could lead to hypotension. The performance of this system had already been analyzed in an observational study *Hatib* [25] and it was shown to predict a hypotensive event within the next 15 s with a likelihood of 85%. This study intended to go further and determine whether the improvement in timely detection would also have an impact on clinical indicators. The median time of hypotension per patient was 8.0 min in the group where the early warning system was implemented versus 32.7 min in the standard care group, being significantly different (P < 0.001).

Anticipating hypoxemia before it occurs would allow anesthesiologists to act proactively in order to prevent hypoxemia and minimize patient harm. With this in mind, *Lundberg et all* [26] presents a machine learning model named, Prescience, that uses standard operating room sensors to predict, in real time during general anesthesia, the risk of hypoxemia and provides explanations of the risk factors. It differs from previous attempts because it provides an explanation of why predictions are made and information on the probable causes in a more clinically relevant way. These explanations are based on information from electronic medical records of more than 50,000 surgeries and are consistent with existing literature and anesthesiologists’ knowledge. The physicians ‘prediction performance with help from Presecience improved from AUC 0.60 to 0.76 (P < 0.0001) for initial risk prediction, and from AUC 0.66 to 0.78 (P < 0.0001) for intraoperative real-time (next 5 min) risk prediction of hypoxemia.

AI application was also studied in the prediction of additional complications, *Peng* [27] evaluated the accuracy and discriminating power of an artificial neural network to predict postoperative nausea and vomiting (PONV). Nausea and vomiting have an incidence of 20–30% in patients under general anesthesia and are associated with several complications, therefore, a model capable of identifying high-risk patients who could benefit from preventive pharmacological interventions can be advantageous. The ANN showed an accuracy of 83.3% using 7 variables—gender, type of surgery, ASA status, duration of anesthesia, smoking habits, history of previous PONV and use of postoperative opioid - as inputs to the prediction. This was the best predictive performance among all the tested models (Naıve Bayesian classifier, logistic regression) with a significantly superior discriminatory power (P < 0.05).

### Drug administration control

The appropriate dosage of drugs during anesthesia is extremely important in order to avoid physiological consequences, such as hypotension, hypertension, hypoxia and arrhythmias that can have a great impact on patients outcomes. In this group, 8 articles were included and are described in Table 5.

**Table 5:** The application of AI in drug administration control

Study	Aim	Population	AI method	N	Accuracy results	Conclusions
Mendez 2018 [28]	Automatic drug delivery	ASA I and II undergoing general anesthesia for ambulatory surgery	FL closed loop system	85	MDAPE 9,67% (AI) vs. 13,5% (control); MDPE − 3,13% vs. -9,83%;	The system showed better performance parameter values than the manual standard procedure and PID algorithms.
Zaouter 2017 [29]	Closed loop sedation without manual override	Patients undergoing elective Transcatheter Aortic Valve Implantation	Hybrid sedation system	20	MDAPE 23,8%; MDPE − 2,4%;	The System maintained sedation in 95% of cases with no manual override.
Xu 2022 [30]	Computer aided diagnosis system on anesthesia quality control	ASA I to III undergoing sedative EGD and/or colonoscopy	CNN	154	Emergence and recovery time were significantly shorter than that in the control group (p < 0.01)	Satisfaction scores were significantly higher and the emergence and recovery time was significantly shorter.
Syed 2021 [31]	Prediction if a colonoscopy can be successfully completed with moderate sedation	Patients undergoing colonoscopy	Machine learning	10.025	AUC 0,762	The model achieved reliable accuracy in predicting procedures.
Wei 2021 [58]	Assessing intrathecal hyperbaric bupivacaine dose during cesarean section	Term parturient (ASA II to III) presented for elective cesarean section under spinal anesthesia	Machine learning	684	MSE 0,0087; Coefficient of determination 0.807	The model created showed a coefficient of determination of 0.807 to predict intrathecal hyperbaric bupivacaine dose
Marrero 2017 [59]	Predict the hypnotic effect in general anesthesia	Patients undergoing general anesthesia using intravenous hypnotic agent (propofol)	FL	20	RMSE for AFM 36,13 vs. 50,29 for the FM vs. 45,69 for the compartmental model.	The model had significantly better results than fuzzy model and compartment model.
Shieh 2000 [60]	Controller for neuromuscular block with rocuronium	ASA I and II undergoing general anesthesia	FL	10	Standard deviation of the T1%² error 1,82% and the mean T1% error − 0,18%.	The performance is better in intermediate duration of action neuromuscular blocking agents.
Lin 2002 [61]	Predict the hypnotic effect of propofol based on clinical parameters	Patients undergoing elective surgery under total intravenous anesthesia	ANN	270	Sn 82,35%; Sp 64,38%; AUC 0.7552	The model had the ability to predict the hypnotic effect of propofol bolus induction superiorly than the clinician.

Mendez et al. [28] has developed an observational study with 81 patients to test a fuzzy logic algorithm with the purpose of controlling propofol infusion and optimal levels of hypnosis (set up as BIS index of 45–55), comparing it with a manual infusion controlled by a senior anesthesiologist. The author claims that his model takes into account all possible complications during surgery - as for hypotension, hypertension - and the correct algorithm to solve them. They reached over 50% of total maintenance time in optimal level of hypnosis without significant adverse effects, overcoming the 37.62% obtained in the control group.

In another perspective, Zaouter et al. [29] created a prospective observational study with the aim of understanding if automated sedation using hybrid sedation systems (HSS) is successful when used in frail and old patients, like those proposed for transcatheter aortic valve implantation (TAVI). This study with 20 patients reveals that robotic sedation with HSS was successful in 95% of the population, meaning that in none of the procedures was necessary manual control by the anesthesiologist. Moreover, none of the patients in the study developed right ventricular failure, which could be a potential complication in this population due to critical respiratory events related to the overshooting of propofol. Nevertheless, the author claims critical respiratory events in 79% of the studied population despite the lower doses of propofol infused and the ability of the robot to decrease the infusion rate by 50%.

Another area where conscious sedation plays a big role is the endoscopic procedures. Cheng Xu et al [30] developed a randomized, single-blinded trial using an AI digestive endoscope that could help improve the quality of sedation during gastrointestinal endoscopic procedures. The trial involved 154 patients, classified with American Society of Anesthesia (ASA) I to III, that were proposed to do endoscopy procedures with ENDOANGEL system – computer-aided quality control system based on deep convolutional neural network models used in parallel with routine endoscopic equipment. This system creates a virtual anatomical model of the gastrointestinal system, showing the areas that still need to be evaluated, reducing the blind spots and the time till the end of the procedure, as it records the examination time and inaccurate scope movement. With this technology the anesthesiologists have a real time controller when to administer or withdraw the medication, improving the induction, emergency and recovery times. Cheng Xu et al. concluded that emergence time and recovery time was shorter in the group of patients with the ENDOANGEL technology, as well the incidence of adverse events - as for cough, hiccup, hypoxemia, hypotension, arrhythmia - although the total dosage of propofol was not statistically different between the two groups. Regardless of being a system designed for gastrointestinal procedures, it allows the anesthesiologists to supplement the dose of anesthetic at proper time, ensuring a proper sedation status.

On another perspective, Syed et al. [31] used a machine learning model to predict the level of sedation required for the endoscopic procedure. This retrospective study, analyzed over ten thousand colonoscopies and concluded that machine learning models can accurately (over 80%) predict which procedures can be successfully done with moderate sedation. Physician performance, total procedure time and patients age were the main influential features.

## Discussion

This study attempted to identify the areas in which AI overlaps clinical anesthesiology, how it can affect its future, the obstacles anesthesiologists must be aware of, and how to approach it. The current literature about AI in clinical practice of anesthesiology is mainly divided in 4 topics: DoA monitoring, image-guided techniques related to Anesthesia, prediction of events/risks related to Anesthesia and drug administration systems.

Most of the current research is at an early stage in the development of AI solutions and focuses primarily on evaluating the accuracy of the models in specific scenarios, achieving promising results. The relevance of DoA monitoring area is related to the quality of the anesthetic protocol being intimately dependent on precise control of the anesthetic target. Finding the optimal form of DoA monitoring is crucial to reduce complications associated with anesthetic overdose (such as cardiac complications, delayed recovery and cognitive dysfunction) without interfering with patient safety. The current state-of-art is largely done by systems based on EEGs, such as BIS, which is the most used system to assess DoA during surgery. It is derived primarily from EEG signals to give a quantitative indication of DoA ranging from 0 to 100. However, it may not be the ideal system, because EEG signals only show the functions of the central nervous system and even in its signals there is a large amount of information present that is not considered into the model, meaning there is possible meaningful data not being fully utilized. Therefore, AI can make a difference by overcoming the limitations of traditional methods and optimizing the already existing advantages. These articles described a lot of different strategies to achieve it, by expansion of the EEG parameters used, covering all its raw possibilities, addition of other clinical monitoring signals capable of maximizing information and optimizing the measurement of anesthetic depth in real time or by finding a new index robust enough to eliminate all the frequently artefactual signals.

Some literature is already a step further and had evaluated the models in a real-world setting, obtaining better outcomes than those of conventional methods, measured by the improvement of clinical parameters. This fact suggests that there is, at least in part, translation of the results obtained in experimental studies to real clinical environments. That’s the case of *Wijnberg’s* [24], *a* randomized control trial, which concluded that a machine learning early warning system had lower median time of hypotension when compared with standard care. In fact, despite being an integral part of healthcare, surgery and anesthesia carry a significant risk of complications and death. The use of AI to identify patients with a higher risk of developing anesthetic complications may shorten the time for medical action, improve therapeutic efficacy and reduce associated morbidities. In this group, we can see two preponderant approaches: some systems have been demonstrated to accurately identify patients at high risk of development of hypotension, hypoxemia, and other conditions; while others showed capability of predicting and alarming that an event is going to happen minutes before it happens. The first one allows for prophylactic measures to be taken in these patients, preventing the development of these complications with a more cost-effective approach. The second one is that if these events do occur, the clinical team will be able to recognize them more quickly, with a more prompt and targeted therapeutic action and, consequently, reducing morbidity and mortality.

In the vast majority, the methods created were able to demonstrate superior results compared to current clinical practice which can be explained by their ability to identify complex nonlinear relationships between dependent and independent variables and finding patterns in complex datasets, with less needing of formal statistical training. One of the great advantages of AI, particularly of deep learning, is that they are capable of selecting themselves, through computational learning, the best possible set of customized features. As a result, the proposed model can uncover complex relations that would not seem obvious when using conventional statistics.

Many technologies have been developed to control drug administration during anesthesia with the aim of adapting the dosage to the patients’ needs and health status. Target control infusion (TCI) was the first step in this direction, but AI models are a potentially game-changing tool that could supplant TCI current performance. This is as a result of its capabilities of integrating many clinical variables as inputs, which makes it possible for the system to make automatic adjustments that are still tailored to the specific needs of the patient at that particular time. This strategy can prevent over or undershooting of drugs, reducing its hemodynamic consequences, and providing the clinician more time to focus on other aspects of the procedure.

The application of AI in health is not restricted to the improvement of traditional clinical methods, but also has the opportunity to boost new ways of providing procedures in cases where traditional one’s don´t extend to. New CNN-based models capable of identifying the best site for neuroaxis blockade are a new solution to patients with specific conditions, especially obese or with spinal pathology. Since it’s a technique requiring high precision, it could be really helpful in certain patients in which the classic method is quite difficult and may even determine the size of the needle that is used. In these cases, AI is not only optimizing a solution, but rather creating a new approach capable of considerable better results. Furthermore, it has the advantage that many models can be developed with different data sets from various institutions that are specific to the characteristics and demographics of their patients. This technological driving force has the potential to standardize globally provided health care by increasing the speed of the experience-efficiency curve of some technical procedures and enabling user-independent levels of acuity that would take a physician years of experience to achieve [19].

In the short to medium term, the idea of AI replacing humans in medicine does not appear to pose a significant threat. In most of the selected papers, AI elements have the primary goal of optimizing skills that require human intervention, restricting themselves to the role of auxiliary tools in a clinical process that is intrinsically human. Within the category of image-guided procedures, the optimization process was centred on enhancing picture quality, identifying elements (vessels/nerves) and instructing or showing the optimal approach, such as the precise puncture location. In this field it becomes explicit that all the optimized competencies have in common the fact they still require a final human intervention. Furthermore, it was humans’ responsibility to set the target that algorithms should be trained for, since the gold standard used in most studies consisted in the opinion of experienced physicians, for example, the labelling of elements in an image, underscoring the co-dependency of AI on human intelligence.

We did find some limitations. Contrary to science-based evidence in the health field, where the size of the population studied is used mostly with the purpose of results analysis, machine learning consumes data to be trained. This data must be only used specifically for this step - the learning of the algorithm. Later its performance needs to be tested on a different set that should be independent enough to discriminating the ability of the resulting decision surface otherwise, we would be inducing a bias called “overfitting” which means we may be overestimating the predictive effect of the model since it was trained and tested on sets with more similarities than the existing ones in real clinical practice. To avoid this, the available data must be divided into a training set used for the learning phase, and a test set for the performance evaluation; most articles used a strategy of splitting data in a 3:1 or 4:1 ratio. Even so, the scarcity of data prevented an extraction of results as robust as desired.

Despite being described as one limitation of the found articles, the lack of enough data is indicative of a structural problem that can be noted as the greatest current barrier to achieving the full potential of AI in health: the lack of a method that enables the collection, storage, and standardization of large-scale data. Today, the healthcare sector generates large quantities of data, but only a fraction of it is accessible for analysis. Prior to concentrating efforts on extracting knowledge from data, it is imperative to define a strategy for how to efficiently collect it. With Europe as the vanguard, many countries are making changes to their laws about data protection and privacy. For example, on 3 May 2022, The European Commission (EC) released a proposal for the European Health Data Space (EHDS), a protocol of a first attempt at a new uniformized and shared system that intends to give researchers access to high-quality health data across borders while protecting patient privacy. This ambitious project, if implemented correctly, might help AI overcome its current bias related to insufficiency data in the health field.

A further limitation of several articles is that they are conducted and evaluated in highly controlled environments and with stringent exclusion criteria that do not permit evaluating the performance of the methods in the face of outliers and the vast clinical diversity that exists in hospital clinical environments. In the bias assessment the principal reason for moderate or high risk was lacking identifying confounding factors and stated strategies to deal with them. This is common to all groups but especially evident around drug administration control, where drug variability was largely ignored as the anesthesia protocol was defined as only a propofol infusion. In the actual surgical environment, patients frequently receive continuous infusions of multiple drugs, such as opioids and muscle relaxants, which can interfere with the hypnotic status, adverse intraoperative events and, ultimately, with drug control infusion. Other limitation is that patients with severe comorbidities were excluded from the greater part of the trials, especially those with kidney or liver disease, which are conditions that can interfere with drug pharmacokinetic and probably would change the developed algorithms, as well as complications during surgery (blood loss for instance) that can have great impact on drug levels, since it causes dynamic and multiple hemodynamic changes. All these compromises the generalization of the results.

“Black box” refers to the difficulty of AI systems, particularly deep learning, to explain the clinical rationale of the reason that leads to their predictions. Intelligent systems can, in fact, recognize patterns and make predictions, but they are incapable of explaining clinical relationships between variables. In a field such as medicine, where it is crucial to understand the physiological concepts underlying a particular intervention in a clinical setting, this constraint has the potential to create trust and transparency issues between the physician and artificial intelligence. There have already been efforts to develop AI capable of explaining its results. *Lundberg et al* [26] machine-learning’s model can help improve the clinical understanding of hypoxemia risk by providing general insights into the precise changes in risk induced by certain patient or procedure characteristics. For instance, it can demonstrate that a patient’s increased risk is attributable to variations in the patient’s tidal volume or pulse rate. This procedure typically implies finding a balance between increasing the interpretability of the predictions and reducing the complexity of the machine-learning model, at the expense of accuracy. However, the authors incorporated in their model recent advances in the area with *“model-agnostic prediction explanation methods”* [32] that allow it to be able to provide theoretically justified explanation without having to reduce its complexity. These advancements have the potential to control the *black box bias*, improving the machine learning suitability in the medical field.

As future challenges, there is a need for further quantitative investigation with larger and more variable datasets, as well as supplementary research focusing on the impact that this application can have on patients’ and physicians’ trust, satisfaction, and eventual moral or ethical dilemmas.

## Conclusion

Early efforts to integrate AI systems into anesthesiology clinical practice have shown promising results and are expected to expand in the near future.

In anesthesiology, it is clear that AI will complement or even replace some of the traditional methods, as a tool to enhance medical professionals’ decision-making skills, diagnostic accuracy and therapeutic response. It is fundamental to establish multidisciplinary collaboration between physicians and data scientists to strengthen the clinical interpretation that is critical for the implementation of this technological transition.

### Electronic supplementary material

Below is the link to the electronic supplementary material.

Supplementary Material 1
