# Supplementary material for: Artificial intelligence and its clinical application in Anesthesiology: a systematic review
Source: J Clin Monit Comput. 2023 Oct 21;38(2):247–59. doi: 10.1007/s10877-023-01088-0 (PMC10995017; doi:10.1007/s10877-023-01088-0)
Supplement: Supplementary file 1 — Supplementary Material 1 [file 10877_2023_1088_MOESM1_ESM.docx]

Artificial Intelligence and its clinical application in Anesthesiology: a systematic review

Supplementary information

| Supplementary Information -Risk of bias of inlcluded studies acording to The Joanna Briggs Institute critical appraisal checklist for analytic cross-section and case-control studies | | | |
| --- | --- | --- | --- |
| Study | Year | Risk of Bias | Q&A |
| Depth of Anesthesia | | | |
| Afshar | 2021 | Moderate | 1-Y; 2-N; 3-Y;4-Y;5-N; 6-U; 7-Y; 8-Y |
| Gu | 2019 | Moderate | 1-U; 2-N; 3-Y; 4-Y; 5-U; 6-U; 7-Y; 8-Y |
| Jiang | 2014 | Moderate | 1-Y; 2-N; 3-Y; 4-Y; 5-N; 6-N; 7-Y; 8-U |
| Lee | 2022 | High | 1-U; 2-N; 3-Y; 4-Y; 5-U; 6-U; 7-Y; 8-U |
| Madanu | 2021 | Moderate | 1-Y; 2-Y; 3- Y; 4-Y; 5-U; 6-U; 7-Y; 8-U |
| Ortolani | 2002 | Low | 1-Y;2-Y; 3-Y; 4-Y; 5-Y; 6-Y; 7-Y; 8-Y |
| Ranta | 2002 | Low | 1-N; 2-Y; 3-Y; 4-Y; 5-Y; 6-Y; 7-U; 8-Y; 9-Y; 10-Y |
| Shalbaf | 2016 | Moderate | 1 -Y; 2-Y; 3-Y; 4-Y; 5-U; 6-U; 7-Y; 8-U |
| Shalbaf | 2018 | Moderate | 1-Y; 2-N; 3-Y; 4-Y; 5-N; 6-N; 7-Y; 8-U |
| Tacke | 2020 | Low | 1-Y; 2-Y; 3-Y; 4-Y; 5-Y; 6-U; 7-Y; 8-U |
| Zhan | 2021 | Low | 1-Y; 2-Y; 3-Y; 4-Y; 5- N; 6- N; 7- Y; 8-Y |
| Zhang | 2000 | Moderate | 1- N; 2 - N; 3-Y; 4-Y; 5-N; 6-N; 7-Y; 8-Y |
| Tosun | 2010 | High | 1-U; 2-U; 3- U; 4-Y; 5-Y; 6-U; 7-U; 8-Y; 9-Y; 10-N. |
| Image-guided techniques | | | |
| Hetherington | 2017 | High | 1-N; 2-N; 3-Y; 4-U; 5-N; 6- N; 7-Y; 8-Y |
| Chan | 2021 | Low | 1-Y; 2-Y;3-Y;4-Y;5-U;6-U;7-Y;8-Y |
| Liu | 2021 | Low | 1-Y; 2-Y; 3-Y; 4-Y; 5-Y; 6-U; 7-U; 8-Y; 9-Y; 10-Y |
| Pesteie | 2017 | Moderate | 1-Y; 2-N; 3-Y; 4-Y; 5-N; 6-N; 7-Y;8-Y |
| Alkhatiba | 2018 | High | 1- N; 2- N; 3- 4- Y; 5-Y; 6-N; 7- N; 8- U |
| Yoo | 2021 | Low | 1 - N; 2 - Y; 3-Y; 4-Y; 5- Y; 6-N; 7-Y; 8-Y |
| Yu | 2014 | Moderate | 1-Y; 2-N; 3-Y; 4-Y; 5-N; 6-N; 7-Y; 8-U |
| Leng | 2016 | Low | 1-Y; 2-Y;3-Y;4-Y;5-Y;6-N;7-Y;8-U |
| Prediction of risk | | | |
| Geng | 2018 | Low | 1-Y; 2-Y; 3-Y; 4-Y; 5-Y; 6-Y; 7-Y; 8-Y |
| Huang | 2000 | Moderate | 1-Y; 2-N; 3-Y; 4-Y; 5-U; 6-U; 7-Y; 8-U |
| Baig | 2012 | Moderate | 1-N; 2-N; 3-Y; 4-Y; 5-U; 6-U; 7-Y; 8-Y |
| Gratz | 2020 | Low | 1-Y; 2-N; 3-Y;4-Y; 5-Y;6-Y; 7-Y; 8-Y |
| Huang | 2022 | Low | 1-Y;2-Y; 3-Y; 4-Y; 5-Y; 6-U; 7-Y; 8-U |
| Knorr | 2006 | High | 1-U; 2-N; 3-Y; 4-Y; 5-N; 6- N; 7-Y; 8-U |
| Lin | 2008 | Low | 1-Y; 2-N; 3-Y; 4-Y; 5-Y; 6-Y; 7-Y; 8-Y |
| Lundberg | 2018 | Low | 1-Y; 2-Y; 3-Y; 4-Y; 5-Y; 6-Y; 7-Y; 8-Y |
| Ren | 2022 | Low | 1-Y; 2-Y; 3-Y; 4-Y; 5-U; 6-N; 7-Y; 8-Y |
| Sippl | 2017 | Moderate | 1-N; 2-N; 3-Y; 4-Y; 5-N; 6-N; 7-Y; 8-Y |
| Zhang | 2018 | Low | 1-Y; 2-Y; 3-Y; 4-Y; 5-U; 6-N; 7-Y; 8-Y |
| Kang | 2020 | Low | 1-Y; 2-Y; 3-Y; 4-Y; 5-Y; 6-Y; 7-Y; 8-Y |
| Kendale | 2018 | Low | 1-Y; 2-Y; 3-Y; 4-Y; 5-U; 6-N; 7-Y; 8-Y |
| Lin | 2011 | Low | 1-Y; 2-Y; 3-Y; 4-Y; 5-N; 6-N; 7-Y; 8-Y |
| Santanen | 2003 | Low | 1-Y; 2-Y; 3-Y; 4-Y; 5-Y; 6-U; 7-Y; 8-Y |
| Wijnberge | 2020 | Low | 1-Y; 2-Y; 3-Y; 4-Y; 5-Y; 6-N; 7-Y; 8-Y |
| Peng | 2007 | Low | 1-Y; 2-Y; 3-Y; 4-Y; 5-N; 6-N; 7-Y; 8-Y |
| Drug Administration | | | |
| Mendez | 2016 | Low | 1-Y; 2-Y; 3-Y; 4-Y; 5-Y; 6-N; 7-Y; 8-Y |
| Zaouter | 2017 | Low | 1-Y; 2-Y; 3-Y; 4-Y; 5-U; 6-U; 7-Y; 8-Y |
| Xu | 2022 | Low | 1-Y; 2-Y; 3-Y; 4-Y; 5-Y; 6-U; 7-U; 8-Y; 9-Y; 10-Y |
| Syed | 2021 | Moderate | 1-Y; 2-Y; 3-U; 4-Y; 5-U; 6-N; 7-Y; 8-Y |
| Wei | 2020 | Moderate | 1-Y; 2-Y; 3-Y; 4-Y; 5-N; 6-N; 7-N; 8-Y |
| Marrero | 2015 | High | 1-N; 2-N; 3-U; 4-Y; 5-N; 6-N; 7-Y; 8-Y |
| Shieh | 2000 | Low | 1-Y; 2-Y; 3-Y; 4-Y; 5-Y; 6-U; 7-Y; 8-Y |
| Lin | 2002 | Moderate | 1-Y;2-Y; 3-U; 4-Y; 5-Y; 6-N; 7-N; 8-Y; 9-Y; 10-U |

*Q&A –* questions and answers, *Y –* yes, *N* – no, *U* – uncertain

**Supplementary Information** - Sources of funding of the studies included in the manuscript

| Study | Year | Source of funding | |
| --- | --- | --- | --- |
| Depth of Anesthesia | | |  |
| Afshar | 2021 | Not discriminated | |
| Gu | 2019 | National Natural Science Foundation of China | |
| Jiang | 2014 | Ministry of Science and Technology, Taiwan | |
| Lee | 2022 | Not discriminated | |
| Madanu | 2021 | Ministry of Science and Technology, Taiwan | |
| Ortolani | 2002 | Not discriminated | |
| Ranta | 2002 | Not discriminated | |
| Shalbaf | 2016 | Not discriminated | |
| Shalbaf | 2018 | Not discriminated | |
| Tacke | 2020 | Departmental sources (KKF) and grant from B. Braun AG Melsungen | |
| Zhan | 2021 | National Key Research and Development Project and Clinical Research Project of Army Medical University | |
| Tosun | 2010 | Not discriminated | |
| Image-guided techniques | | |  |
| Hetherington | 2017 | Natural Sciences and Engineering Research Council of Canada and the Canadian Institutes of Health Research | |
| Chan | 2021 | Ministry of Health’s National Medical Research Council | |
| Liu | 2021 | Not discriminated | |
| Pesteie | 2017 | Natural Scienece and Engineering Research Council of Canada and Canadian Institutes of Health Research | |
| Alkhatib | 2018 | Centre Val de Loire Region (France) grant 2016 | |
| Yoo | 2021 | National Research Foundation of Korea Grant | |
| Yu | 2014 | Not discriminated | |
| Yusong | 2016 | Singhealth Foundation - National Healthcare Innovation Centre Grant | |
| Prediction of risk | | |  |
| Geng | 2018 | No funding | |
| Huang | 2003 | China Postdoctoral Science Foundation and National Natural Science Foundation of China | |
| Baig | 2012 | Not discriminated | |
| Gratz | 2020 | Caretaker | |
| Huang | 2022 | Research Funding Project of Panzhihua University | |
| Knorr | 2006 | Not discriminated | |
| Lin | 2008 | National Science Council of Taiwan | |
| Lundberg | 2018 | National Science Foundation grant, National Institutes of Health grant, NSF Graduate Research Fellowship grant and a UW eScience/ITHS seed grant Machine Learning in Operating Rooms. | |
| Ren | 2022 | Not discriminated | |
| Sippl | 2017 | Not discriminated | |
| Kang | 2020 | Ministry of Education, Soonchunhyang University Research Fund and the National Research Foundation of Korea grant | |
| Kendale | 2018 | Institutional and/or departmental sources | |
| Lin | 2011 | National Science Council of Taiwan | |
| Santanen | 2003 | Not discriminated | |
| Wijnberge | 2020 | Edwards Lifesciences | |
| Peng | 2007 | Not discriminated | |
| Hatib | 2018 | Edwards Lifesciences | |
| Drug Administration | | |  |
| Mendez | 2016 | Spanish Ministry of Education, Culture and Sport | |
| Zaouter | 2017 | Department of Anesthesia and Critical Care of the CHU de Bordeaux | |
| Xu | 2022 | Hubei Province Major Science and Technology Innovation Project and Independent research project of Wuhan University | |
| Syed | 2021 | Translational Research Institute | |
| Wei | 2020 | Technology Bureau of Jiaxing, Zhejiang Province and the Key Discipline established with Zhejiang Province and Jiaxing City jointly | |
| Marrero | 2015 | Ministerio de Ciencia e Innovación | |
| Shieh | 2000 | Not discriminated | |
| Lin | 2002 | Not discriminated | |
| Miscelaneous | | | |
| MacCarthy | 2004 | Not discriminated | |
| Rezayi | 2022 | Not discriminated | |
| Char | 2020 | No funding | |
| Chae | 2020 | Not discriminated | |
| Singh | 2022 | No funding | |
| Zaouter | 2020 | No funding | |
| Jin | 2020 | y National Natural Science Foundation of China | |
| Bedrikovetski | 2021 | Colorectal Surgical Society of Australia and New Zealand | |
| Li | 2022 | Radiological Society of North America, GE Healthcare and Genentech Foundation | |
| Murray | 2020 | No funding | |
| Goldstein | 2021 | No funding | |
| Hashimoto | 2020 | Institutional and/or departmental sources | |
| Liang | 2020 | National Natural Science Foundation of China and Natural Science Fund for Excellent Young Scholars of Hebei Province of China | |
| Štrumbelj | 2014 | Not discriminated | |
| Bainbridge | 2008 | Not discriminated | |
| Hu | 2017 | National Natural Science Foundation of China | |
